# Factors Associated with Infection Control Competency Among Infection Control Nurses and Physicians in Long-Term Care Hospitals: A Cross-Sectional Study

**DOI:** 10.3390/healthcare14121709

**Published:** 2026-06-15

**Authors:** Jeong Sil Choi, Kyung Mi Kim

**Affiliations:** 1 College of Nursing, Gachon University, Incheon 21936, Republic of Korea; jschoi408@empas.com; 2 College of Nursing, Chungbuk National University, Cheongju 28644, Republic of Korea

**Keywords:** infection control, long-term care, nurses, physicians

## Abstract

Background: The number of long-term care hospitals (LTCHs) in Korea has been increasing. Because LTCHs primarily care for older adults who are vulnerable to infection, strict infection control (IC) practices are essential. This study aimed to assess the IC core competency of infection control nurses (ICNs) and IC physicians in LTCHs and to identify the individual and organizational factors associated with IC core competency. Methods: A descriptive cross-sectional survey was conducted among 348 participants (273 ICNs and 75 IC physicians) working in LTCHs across Korea in December 2022. Data were analyzed to assess levels of IC core competency, IC knowledge, and organizational support for patient safety. Results: The overall scores for IC core competency, IC knowledge, and organizational support for patient safety were 3.2 ± 0.7, 3.3 ± 0.7, and 3.4 ± 0.7 (out of 5), respectively. For ICNs, factors associated with IC core competency included IC nurse specialist certification, pre-employment IC training, attendance at IC-related academic conferences or training courses, IC knowledge, and organizational support for patient safety. For IC physicians, attending IC-related academic conferences or training courses, IC knowledge, and organizational support for patient safety were identified as significant factors associated with competency levels. Conclusions: This study found that IC knowledge, IC core competency, and organizational support for patient safety were insufficient among ICNs and IC physicians in LTCHs. IC Knowledge was the factor most strongly associated with IC core competency, and organizational support for patient safety also emerged as an important associated factor.

## 1. Introduction

The incidence of healthcare-associated infections (HAIs) continues to rise, driven by the growing number of elderly and immunocompromised patients, the emergence of antibiotic-resistant organisms, and the increased use of invasive diagnostic and therapeutic procedures. The number of long-term care hospitals (LTCHs) in Korea is increasing continuously due to the rapid increase in the elderly population. In Korea, long-term care (LTC) is provided through two distinct systems, LTC Insurance and National Health Insurance (NHI), which cover nursing homes (NHs) and LTCHs, respectively [1]. Reflecting this institutional distinction, LTCHs provide long-term inpatient care centered on medical and nursing services for older adults, patients with chronic diseases, and individuals requiring LTC and treatment whereas NHs primarily provide assistance with daily living within the LTC system [1]. Because many patients in LTCHs have prolonged hospital stays, they are increased risk of infection. Outbreaks of infections in LTCHs have been repeatedly documented [2], including during the COVID-19 pandemic, which further emphasizes the critical need for effective infection control (IC) practices in these settings.

To strengthen IC, the Korean Medical Act mandates the establishment of IC committees and departments in general hospitals with more than 150 beds, including the assignment of one infection control nurse per 150 beds and annual completion of 16 h of IC education for both infection control nurses (ICNs) and IC physicians. However, prior to the revision, LTCHs had largely remained outside the scope of these regulations. Following the COVID-19 pandemic, the Medical Act was revised in December 2021 to include hospitals with 100 or more beds, thereby extending these IC requirements to many LTCHs for the first time. Despite these policy changes, previous studies have revealed significant gaps in IC infrastructure at LTCHs in Korea. For example, only 17.4% of LTC hospitals had dedicated IC departments [1], and merely 5.4% had designated IC professionals [3]. Similar trends have been observed internationally. In the United States, for instance, only 2.7% of IC personnel in nursing homes hold certification, and over half manage multiple roles without specialized IC training [4].

ICNs at LTCHs in Korea have reported insufficient job competency, a lack of IC personnel, and inadequate support systems [5]. Among the 900 nursing homes in the United States, 84% of IC practitioners were ICNs, with 54% performing at least two roles, and 61% working without having undergone specific IC training [6]. In another study of 922 nursing homes, only 38.9% of IC practitioners had received IC training, and only 2.7% were IC-certified, highlighting the necessity of educating and training IC practitioners in nursing homes [4]. In a recent study [7] conducted in Florida among infection prevention and control personnel in LTC facilities (LTCFs), 64.3% of infection preventionists had fewer than 5 years of experience. Similarly, in our study, 16.7% of IPs had less than 1 year of experience in the field. Taken together, these findings underscore the urgent need for stronger training and support systems, including continuous professional development opportunities and peer mentorship. Given that ICNs and IC physicians are responsible for implementing and overseeing IC prevention programs, their core competencies are essential for ensuring patient safety [8].

Organizations such as the Association for Professionals in Infection Control and Epidemiology (APIC), the Community and Hospital Infection Control Association-Canada (CHICA-Canada), and the Australian Infection Control Association (AICA) have conducted studies on competencies and practice standards in the IC professionals [8,9,10]. However, few studies have assessed the core competencies of IC personnel in LTC settings or investigated the factors associated with these competencies. Some studies have identified IC certification and experience as significant factors related to IC practices [11,12]. However, further research is needed to explore the core IC competencies and the factors associated with these competencies among ICNs and IC physicians, who typically serve as institutional representatives in LTCHs, such as LTCHs and nursing homes in Korea. Such studies should incorporate objective performance-based assessments to complement the current findings and bridge the gap between perceived confidence and clinical expertise. The evaluation of IC core competencies should encompass both individual characteristics, such as knowledge, and organizational factors, including organizational support [11,12,13].

Organizational support encompasses practical management strategies, including the influence on work processes and design, as well as human resource policies [13]. IC is closely linked to patient safety and is influenced by the extent of organizational support. This support impacts both job satisfaction and burnout among ICNs [14]. These factors, in turn, can affect ICN turnover rates, highlighting the importance of understanding the level of organizational support necessary for effective infection control programs.

Given the high-risk population served by LTCHs, it is crucial to enhance the core competencies of ICNs and IC physicians. Therefore, this study aims to assess the IC core competencies of ICNs and IC physicians in LTCHs in Korea, utilizing the framework established by the Certification Board of Infection Control (CBIC) [15]. Additionally, the study seeks to identify individual and organizational factors that influence these competencies. The findings will provide insights into strategies to strengthen IC capacity and infrastructure within LTC settings.

## 2. Methods

### 2.1. Study Design

This study utilized a descriptive cross-sectional survey design to assess the core competencies of ICNs and IC physicians in LTCHs in Korea and to identify factors associated with these competencies.

### 2.2. Participants and Data Collection

Eligible participants were ICNs and IC physicians responsible for IC practices in LTCHs, who understood the purpose of the study and provided informed consent. Individuals not directly involved in IC or those refusing participation were excluded.

The sample size calculation was based on a total of 1451 LTCHs registered in Korea as of August 2022. Assuming a 95% confidence level and a margin of error of ±5%, the minimum number of participants required for this study was estimated to be 304 [16].

Participants were recruited in collaboration with the Korea Human Resource Development Institute for Health and Welfare and the Korean Association of Infection Control Nurses (KAICN). Recruitment was conducted through the dissemination of research announcements posted on the websites of these organizations. Additionally, emails containing detailed explanations of the study, along with online consent forms and a survey link via Google Forms, were sent to IC personnel who had received IC training through these organizations. The survey was distributed and collected via email over a one-month period in December 2022. A total of 550 individuals accessed the email, with 375 providing responses (response rate of 68.2%). After excluding 27 participants who provided incomplete or unreliable responses or were not ICNs or IC physicians, data from 348 participants (273 ICNs and 75 IC physicians) were included in the final analysis.

### 2.3. Measurements

#### 2.3.1. Participant and Organizational Characteristics

Participant and organizational characteristics were assessed using an instrument originally developed by Choi [11] and Kim and Choi [12] to evaluate the competency of ICNs in general hospitals in Korea. Participant characteristics included gender, age, total clinical experience, duration of IC experience, current job position, educational background, possession of ICN specialist certification, completion of IC training prior to assuming an IC position, participation in continuing education after employment, and annual attendance at conferences or training courses. Organizational characteristics included hospital location, bed capacity, and type of infection control activities performed.

#### 2.3.2. Perceived Knowledge of IC

The perceived level of IC knowledge among ICNs and IC physicians in LTCHs was assessed using an instrument developed by Choi and Kim [15], based on the ICNs’ competency proposed by the CBIC in 2012. This instrument comprises nine items covering subdomains such as identification of the infectious disease process, surveillance and epidemiologic investigations, prevention and control of the transmission of infectious agents, employee and occupational health, management and leadership, communication and feedback, quality and performance improvement, patient safety, education and research. This instrument is a self-assessment measure of perceived level of IC nowledge. Each item was rated on a 5-point Likert scale, ranging from 1 (“very insufficient”) to 5 (“very sufficient”), with higher scores indicating greater perceived IC knowledge. The Cronbach’s alpha for this instrument was 0.92 in the study by Choi and Kim [14] and 0.92 in the present study. To ensure the content validity and applicability of the instrument in the LTCHs setting, the items were reviewed by an expert panel consisting of two nursing professors and three ICNs with extensive experience in LTC facilities. In addition, a pilot test was conducted with five IC practitioners (three ICNs and two physicians) working in LTCHs to evaluate the clarity and relevance of the items. Minor modifications were made to the wording to better reflect the LTCHs context.

#### 2.3.3. IC Core Competency

IC core competency was assessed using a 47-item instrument developed by Kim and Choi [12] based on the core competencies for ICNs proposed by the CBIC in 2012. Participants rated their perceived level of core competency on a 5-point Likert scale, ranging from “not performing at all” to “not performing well,” “moderate,” “performing well,” and “performing very well.” Higher scores indicate greater competency in IC. The Cronbach’s alpha for this instrument was 0.96 in the study by Kim and Choi [12] and 0.98 in the present study.

#### 2.3.4. Organizational Support for Patient Safety

Organizational support for patient safety was assessed using a seven-item instrument, with all items extracted from the Hospital Survey on Patient Safety Culture developed by the Agency for Healthcare Research and Quality (AHRQ) [13]. The instrument comprises three items from the “Management Support for Patient Safety” domain, one item from the “Overall Perceptions of Patient Safety” section, and three items from the “Staffing” domain.

Content validity of the instrument was reviewed by two nursing professors and two certified ICNs. Each item was rated on a 5-point Likert scale, ranging from 1 (“strongly disagree”) to 5 (“strongly agree”), with higher scores indicating greater perceived organizational support for patient safety. In the present study, the Cronbach’s alpha for this instrument was 0.89.

### 2.4. Data Analysis

Data were analyzed using SPSS for Windows version 26.0 (IBM Corp., Armonk, NY, USA). Descriptive statistics, including frequencies, percentages, mean and standard deviation, were used to summarize participant and organizational characteristics. The normality of the core competency scores was assessed using normality tests. Pearson’s correlation coefficients were calculated to examine relationships between continuous variables. Hierarchical multiple regression analysis was performed to identify factors associated with IC core competency.

## 3. Results

### 3.1. Personal and Hospital Characteristics of the Participants

A total of 348 participants were included, consisting of 273 ICNs and 75 IC physicians. The hospitals where the participants were employed had an average bed capacity of 207.7, with the largest proportion of participants (48.3%, *n* = 168) working in hospitals with 100–200 beds. Among the ICNs, 97.4% (*n* = 226) were women, and the mean age was 50.6. The average duration of experience in IC was 2.4 years, with 35.9% (*n* = 98) reporting less than one year of experience. Most ICNs held positions as head nurses or managers (74.8%), and 95.2% (*n* = 260) held dual roles. Regarding educational background, 49.8% (*n* = 136) held an associate degree (a 3-year college program in Korea), and 92.7% did not possess an ICN specialist certification. Before assuming their IC role, 48.0% (*n* = 131) had received IC training. After starting their IC roles, 78.4% (*n* = 214) complied with the 16-h mandatory continuing education requirement, whereas 60.4% did not attend IC-related academic conferences or training courses annually.

For the IC physicians, 88.0% (*n* = 66) were men, with a mean age of 55.5 years. The average duration of IC experience was 1.8 years, and 40.0% (*n* = 30) had less than one year of experience. Most IC physicians (60.0%) were hospital directors, and almost all (98.7%, *n* = 74) held dual roles. Regarding educational level, 44.0% (*n* = 33) had a bachelor’s degree, and 92.0% (*n* = 69) were certified medical specialists. Before starting their IC roles, 66.7% (*n* = 50) had not received IC training, and 73.3% (*n* = 55) completed the 16-h mandatory training for continuing education after assuming IC roles, whereas 54.7% (*n* = 41) did not attend IC-related conferences or training courses annually (Table 1).

### 3.2. Main Continuous Variables

The mean IC core competency score for all participants was 3.2 ± 0.7 (on a 5-point scale). The mean IC core competency scores were 3.1 ± 0.7 for ICNs and 3.6 ± 0.7 for IC physicians.

Among the subdomains of IC core competency, “prevention and control of transmission of infectious agents” had the highest score at 3.5 ± 0.7, while “research” scored the lowest at 2.8 ± 1.0 (Table 2).

The overall mean knowledge score for IC was 3.3 ± 0.7. Specifically, the mean knowledge scores were 3.1 ± 0.7 for ICNs and 3.6 ± 0.6 for IC physicians. Within the knowledge subdomains, “employee/occupational health” received the highest score (3.5 ± 0.8), and “research” received the lowest (2.6 ± 1.0) (Table 2).

The mean organizational support for patient safety (on a 5-point scale) was 3.4 ± 0.7 overall. For ICNs and IC physicians, these scores were 3.3 ± 0.7 and 3.7 ± 0.6, respectively (Table 2).

### 3.3. Correlation Between IC Core Competency and Main Continuous Variables

Among ICNs, IC core competency was significantly positively correlated with age, clinical experience, IC experience, knowledge of IC, and organizational support for patient safety (*p* < 0.05). Among IC physicians, IC core competency was significantly positively correlated with IC experience, knowledge of IC, and organizational support for patient safety (*p* < 0.05) (Table 3).

### 3.4. Factors Associated with the IC Core Competencies of ICNs and IC Physicians

Regarding individual characteristics, ICN specialist certification, and education-related variables (pre-employment IC training, continuing education after starting IC work, attendance at IC-related academic conferences or training courses), which had shown significant association in previous studies and in the correlation analysis, were entered at Step 1 [17,18,19].

At Step1, ICN specialist certification (β = 0.173, *p* = 0.003), pre-employment training (β = 0.134, *p* = 0.027), and attendance at IC-related academic conferences or training courses (β = 0.141, *p* = 0.020) were significantly associated with core competency, accounting for 12.5% of the variance in IC core competency scores. At Step 2, knowledge of IC was significantly associated with IC core competency (β = 0.643, *p* < 0.001), accounting for an additional 36.5% of the variance. At Step 3, organizational support for patient safety was also significantly associated with IC core competency (β = 0.215, *p* < 0.001), accounting for an additional 3.7% of the variance in IC core competency. The final model explained 52.7% of the total variance in core competency among ICNs.

For IC physicians, only attendance at IC-related academic conferences or training courses (β = 0.235, *p* = 0.049) was significantly associated with IC core competency at Step 1, accounting for 10.4% of the variance. At Step 2, knowledge of IC was significantly associated with core competency (β = 0.803, *p* < 0.001), accounting for 57.9% of the variance. At Step 3, organizational support remained significantly associated with IC core competency factor (β = 0.288, *p* < 0.001), accounting for an additional 5.1% of the variance. The final model explained 73.4% of the total variance in IC core competency among IC physicians.

The regression analysis met the basic assumptions for independence of errors and multicollinearity. The Durbin-Watson statistics were 1.996 for ICNs and 1.995 for IC physicians, indicating no evidence autocorrelation. Furthermore, no multicollinearity was observed, as the variance inflation factor (VIF) values ranged from 1.054 to 1.227 for ICNs and from 1.094 to 1.374 for IC physicians, all of which were well below the threshold of 10 (Table 4).

## 4. Discussion

In Korea, most patients admitted to LTCHs are elderly individuals who require rehabilitation or assistance with activities of daily living due to conditions such as Parkinson’s disease, dementia, cerebrovascular diseases, cancer, or diabetes [3]. These patients are particularly vulnerable to infection. Furthermore, frequent transfers between acute care hospitals for advanced treatment and subsequent readmission to LTCHs can facilitate the transmission of multidrug-resistant organisms. Therefore, the implementation of IC practices comparable to those in acute care hospitals is essential in LTCHs as well [3]. However, before the recent revision of relevant policies, IC systems in Korean LTCHs were not well established, and institutional support for dedicated IC activities remained limited [3]. As a result, the COVID-19 pandemic revealed persistent issues in IC, including frequent outbreaks, in LTCHs. Since 2022, LTCHs have also been required to designate ICNs and IC physicians, as well as to establish IC committees and departments, in order to receive the government-designated IC fee for COVID-19 prevention and management. This policy change has led to a significant increase in the number of ICNs and IC physicians employed in LTCHs [19].

In this study, ICNs and IC physicians working in LTCHs in Korea had a mean of 18 years of clinical experience, but fewer than three years of IC experience. This may reflect the relatively recent implementation of formal IC systems in Korean LTCHs. Because LTCHs were not previously subject to the same level of formal requirements for IC infrastructure and personnel, opportunities to accumulate IC experience may have been limited. Given this context, the limited IC experience observed in our sample may help explain the relatively low levels of IC competency. This interpretation is consistent with international studies in LTC settings, which have described limited IC and prevention training, limited experience, multiple role assignments among personnel responsible for infection prevention, and constraints in implementing infection prevention programs [5,7,20].

In our study, ICNs and IC physicians in LTCHs demonstrated relatively low level of IC core competency. In particular, educational competency was the lowest among IC physicians, while research competency was the lowest among ICNs. As most IC physicians in LTCHs hold the position of hospital director, they may feel a responsibility to lead IC education within their institutions. However, due to the lack of a background in infection-related specialties, many IC physicians reported insufficient knowledge of IC guidelines, which contributed to their limited educational competency. In our study, research competency was the lowest domain among ICNs in LTC hospitals, and their knowledge related to research was also limited. This may reflect the reality that research is rarely prioritized as a primary component of IC roles in LTC settings. These findings are consistent with previous studies [12,13]. In particular, using the same assessment tool, also reported that research competency was the lowest among ICNs in Korean hospitals with more than 300 beds. While surveillance is considered an essential task in acute care hospitals, surveillance competency among ICNs in LTCHs was substantially lower than that reported by Choi and Kim [15]. Surveillance and epidemic investigation are central responsibilities of IC personnel; however, in Korean LTCHs, directors of nursing or head nurses are often assigned IC duties in addition to their primary roles, which can hinder systematic IC practice. In the United States, surveillance, along with investigations into antibiotic use and staff education, has been identified as one of the tasks requiring the greatest time investment for IC professionals [6]. However, in the present study, this lower level of surveillance competency among ICNs in LTCHs is likely attributable to the fact that most ICNs work in dual roles, and only about 61.7% were directly involved in surveillance activities [18].

In contrast to other countries, Korean regulations require IC personnel in hospitals to complete annual IC training, as stipulated by the Medical Act. Under these regulations, approximately 4000 IC personnel in LTCHs receive such training annually [19]. In this study, participating in IC-related academic conferences or training courses, as well as possessing greater IC knowledge, were identified as factors associated with the IC core competencies of ICNs and IC physicians in LTCHs. Therefore, the development of a comprehensive, career-level IC program for IC personnel at the national level is warranted.

In this study, organizational support for patient safety emerged as a significant determinant of IC competency, although the overall perceived level of such support was not high. Notably, IC physicians reported higher levels of organizational support than ICNs, which may be explained by the fact that many physician respondents were hospital directors. As individuals in leadership positions, they may perceive institutional commitment more positively than frontline IC practitioners. This finding is particularly meaningful in Korean LTCHs, where the role of organizational support may differ from that in acute care hospitals. In acute care hospitals, where IC systems and infrastructures are generally more established, organizational support may function primarily as a facilitating contextual factor. In contrast, many Korean LTCHs operated under voluntary IC practices prior to the 2021 revision of the Medical Act [21], and thus have historically faced more limited institutional requirements and fewer structural resources for IC. In such settings, organizational support may play a different role than in general hospitals; it acts as a fundamental top-down mechanism that bridges the gap between individual knowledge and actual clinical practice. Without management’s commitment to providing isolation facilities, reimbursement for disposables, and sufficient staffing [5], ICNs may face structural barriers that limit the effective use of their expertise. Previous studies have reported that the level of patient safety culture perceived by ICNs working in LTCHs in Korea is lower than that in general or specialized hospitals [21]. Therefore, the significant influence of organizational support found in this study underscores that improving IC competency in LTC hospitals requires not just individual training but a systemic shift in leadership awareness and a non-punitive safety culture [22].

In this study, both ICNs and IC physicians in LTCHs demonstrated a significant positive relationship between IC core competency and organizational support for patient safety. Organizational support for patient safety was identified as a significant factor associated with IC core competency in both groups, highlighting the importance of hospital management’s commitment and support. Furthermore, previous research among ICNs has shown that organizational support is statistically correlated with IC knowledge, attitudes, and practices [23]. A positive patient safety culture has also been identified as a predictive factor for the incidence of major HAIs and employee safety [23]. Consequently, fostering an organizational culture that supports IC within hospitals is crucial for the prevention of HAIs.

This study has several limitations. First, the assessment of IC core competency and perceived knowledge was based on self-reported measures, which may be subject to social desirability bias and may not fully reflect actual proficiency. Second, as the survey was conducted among ICNs and IC physicians in LTCHs in Korea, the findings may not be generalizable to other contexts. Third, participants were recruited through professional organizations and training-related platforms, and participation was voluntary; therefore, the sample may have been subject to self-selection bias and may not fully represent all IC personnel working in LTCHs. Future research incorporating objective assessment methods, such as standardized knowledge tests or direct clinical observation, is warranted to provide a more comprehensive evaluation of IC proficiency in LTCHs.

## 5. Conclusions

This study aimed to identify the general characteristics of ICNs and IC physicians in LTCHs, to assess their levels of IC knowledge, IC core competency, and organizational support for patient safety, and to analyze factors associated with their IC core competencies. The results indicated that both ICNs and IC physicians in LTCHs exhibited insufficient levels of IC core competency and knowledge. Among the factors identified, IC knowledge was most strongly associated with IC core competency, and regular attendance at IC-related academic conferences was also a significant factor related to competency levels.

These findings highlight the need for systematic and regular IC education programs tailored to the competency levels of ICNs and IC physicians in LTCHs. Moreover, although the perceived level of organizational support for patient safety was not high, it emerged as a significant organizational factor associated with IC core competency.

Therefore, national-level support to enhance the IC infrastructure and organizational culture in LTCHs, where vulnerable populations are frequently hospitalized, is essential to strengthen patient safety. Ultimately, improving these areas will help achieve the primary goal of IC, which is the safety of patients.

## Figures and Tables

**Table 1:** Personal and hospital characteristics (N = 348).

Variable	Category	Total (*N*)	%	ICNs (*n* = 273)		IC Physicians (*n* = 75)	
				*n*	%	*n*	%
Personal Characteristics							
Gender	Women	275	79.0	266	97.4	9	12.0
	Men	73	21.0	7	2.6	66	88.0
Age (years)	M ± SD	51.614 ± 10.37		50.55 ± 9.58		55.45 ± 12.16	
Clinical experience (years)	M ± SD	17.80 ± 11.94		17.57 ± 11.54		18.63 ± 14.23	
Infection control experience (years)	<1	128	36.8	98	35.9	30	40.0
	1–<3	114	32.8	85	31.1	29	38.7
	3–<5	59	17.0	49	17.9	10	13.3
	5–<7	26	7.5	21	7.7	5	6.7
	≧7	21	6.0	20	7.3	1	1.3
	M ± SD	2.26 ± 2.48		2.39 ± 2.66		1.78 ± 1.63	
Position	Staff nurse	34	9.8	34	12.5	-	-
	Charge nurse	14	4.0	14	5.1	-	-
	Head nurse	99	28.4	99	36.3	-	-
	Manager	133	38.2	105	38.5	28	37.3
	Head of department	17	4.9	15	5.5	2	2.7
	Top manager	51	14.7	6	2.2	45	60.0
Degree	3-year College	136	39.1	136	49.8	-	-
	Bachelor	129	37.1	96	35.2	33	44.0
	Master’s	64	18.4	40	14.7	24	32.0
	Doctoral	19	5.5	1	0.4	18	24.0
Infection control nurse specialist certification	No	322	92.5	253	92.7	69	92.0
	Yes	26	7.5	20	7.3	6	8.0
Pre-education	Unlessoned	192	55.2	142	52.0	50	66.7
	Lessoned	156	44.8	131	48.0	25	33.3
Continuing education after starting infection control work	Not educated	6	1.7	5	1.8	1	1.3
	Partially educated	73	21.0	54	19.8	19	25.3
	Accurately educated	269	77.3	214	78.4	55	73.3
Attendance of academic conferences or training courses (every year)	Non-attendance	206	59.2	165	60.4	41	54.7
	Attendance	142	40.8	108	39.6	34	45.3
Hospital characteristics							
Hospital location	Province	229	65.8	179	65.6	50	66.7
	Metropolitan city	119	34.2	94	34.4	25	33.3
Hospital bed	<100	27	7.8	23	8.4	4	5.3
	>100–<200	168	48.3	129	47.3	39	52.0
	>200–<300	112	32.2	87	31.9	25	33.3
	≧300	41	11.8	34	12.5	7	9.3
	M ± SD	207.71 ± 87.84		209.19 ± 88.52		202.55 ± 85.71	
Working condition	Dual role	334	96.0	260	95.2	74	98.7
	Full-time	14	4.0	13	4.8	1	1.3

ICNs = Infection Control Nurses; IC physicians = Infection Control physicians.

**Table 2:** Level of core competencies and knowledge and organizational support for patient safety in ICNs and IC physicians (N = 348).

Variables (Range)	Total	ICNs (*n* = 273)	IC Physicians (*n* = 75)
		M ± SD	M ± SD
**Core competencies of infection control and prevention** **identified by CBIC** (scores of 1–5)	3.2 ± 0.7	3.1 ± 0.7	3.6 ± 0.7
Identification of infectious disease processes	3.2 ± 0.8	3.0 ± 0.8	3.8 ± 0.8
Sureillance and epidemiologic investigations	2.9 ± 0.9	2.8 ± 0.9	3.4 ± 0.8
Preventing/controlling the transmission of infectious agents	3.5 ± 0.7	3.5 ± 0.7	3.8 ± 0.7
Employee/occupational health	3.3 ± 0.9	3.2 ± 0.9	3.6 ± 0.8
Management and leadership	3.1 ± 0.9	3.0 ± 0.9	3.5 ± 0.8
Communication and feedback	3.3 ± 0.9	3.2 ± 0.9	3.6 ± 0.8
Quality/performance improvement and patient safety	3.3 ± 0.9	3.2 ± 0.9	3.6 ± 0.8
Education	3.0 ± 0.9	2.9 ± 0.9	3.3 ± 0.9
Research	2.8 ± 1.0	2.7 ± 0.9	3.4 ± 0.9
**Knowledge of infection control (scores of 1–5)**	3.3 ± 0.7	3.1 ± 0.7	3.6 ± 0.6
Identification of infectious disease processes	3.3 ± 0.8	3.2 ± 0.8	3.6 ± 0.7
Surveillance and epidemiologic investigations	3.1 ± 0.9	3.0 ± 0.9	3.5 ± 0.8
Preventing/controlling the transmission of infectious agents	3.3 ± 0.8	3.3 ± 0.8	3.7 ± 0.8
Employee/occupational health	3.5 ± 0.8	3.5 ± 0.8	3.8 ± 0.8
Management and leadership	3.4 ± 0.9	3.4 ± 0.9	3.8 ± 0.8
Communication and feedback	3.5 ± 0.8	3.4 ± 0.8	3.7 ± 0.8
Quality/performance improvement and patient safety	3.4 ± 0.8	3.3 ± 0.8	3.8 ± 0.7
Education	3.4 ± 0.9	3.3 ± 0.9	3.7 ± 0.9
Research	2.6 ± 1.0	2.5 ± 1.0	3.0 ± 1.1
**Organizational support for Patient Safety (scores of 1–5)**	3.4 ± 0.7	3.3 ± 0.7	3.7 ± 0.6
Hospital management provides a work climate that promotes patient safety	3.7 ± 0.9	3.7 ± 0.9	4.0 ± 0.8
The actions of hospital management show that patient safety is a top priority	3.8 ± 1.0	3.7 ± 1.0	4.1 ± 0.8
Hospital management seems interested in patient safety only after an adverse event happens (R)	3.7 ± 1.0	3.5 ± 1.0	4.0 ± 0.8
Our procedures and systems are good at preventing errors from happening	3.5 ± 0.9	3.4 ± 0.9	3.8 ± 0.8
We have enough staff to handle the workload	3.2 ± 1.0	3.1 ± 1.1	3.7 ± 0.9
Staff in this unit work longer hours than is best for patient care (R)	3.2 ± 1.0	3.1 ± 1.0	3.7 ± 0.9
We work in “crisis mode” trying to do too much, too quickly (R)	2.9 ± 1.0	3.0 ± 0.7	2.9 ± 1.1

CBIC, Certification Board of Infection Control and Epidemiology; ICNs, Infection Control Nurses; IC physicians, Infection Control Physicians; R, Rerverse-worded item.

**Table 3:** Correlations between variables and infection control core competencies of ICNs and IC physicians (N = 348).

	Core Competencies	
Variables	ICNs (*n* = 273) r (*p*)	IC Physicians (*n* = 75) r (*p*)
Hospital beds	−0.05 (0.429)	0.02 (0.858)
Age (years)	0.21 (<0.001 *)	0.10 (0.403)
Clinical experience (years)	0.14 (0.022 *)	0.19 (0.097)
Infection control experience (years)	0.18 (0.004 *)	0.25 (0.029 *)
Knowledge of infection control	0.71 (<0.001 *)	0.82 (<0.001 *)
Organizational support	0.47 (<0.001 *)	0.69 (<0.001 *)

Pearson’s correlation coefficients. * *p* < 0.05. ICNs, Infection Control Nurses; IC physicians, Infection Control physicians.

**Table 4:** Results of hierarchical multiple regression analysis for IC core competencies (N = 348).

Variables	ICNs (*n* = 273)				IC Physicians (*n* = 75)			
	β	*p*	R^2^ Change	Adjusted R^2^	β	*p*	R^2^ Change	Adjusted R^2^
Level 1			0.125	0.125 **			0.104	0.104 *
Age (years)	0.102	0.141			0.096	0.486		
Clinical experience (years)	−0.001	0.987			0.101	0.450		
Infection control experience (years)	0.094	0.122			0.194	0.097		
Infection control specialist certification	0.173	0.003			0.027	0.813		
Pre-education	0.134	0.027			0.045	0.697		
Continuing education after starting infection control work	0.117	0.051			0.168	0.154		
Attendance of academic conferences or training courses (every year)	0.141	0.020			0.235	0.049		
Level 2			0.365	0.490 **			0.579	0.683 **
Knowledge of infection control	0.643	<0.001			0.803	<0.001		
Level 3			0.037	0.527 **			0.051	0.734 **
Organizational support	0.215	<0.001			0.288	<0.001		

Computed by independent hierarchical multiple linear regression analysis. * *p* < 0.05, ** *p* < 0.01. Durbin-Waston; ICNs = 1.996, IC physicians = 1.995. VIF (variance inflation factor); ICNs 1.054–1.227, IC physicians 1.094–1.374. Dummy variables (infection control specialist certification, pre-education, attendance of academic conference or training course; yes = 1, continuing education after starting infection control work; accurately educated = 1). ICNs, Infection Control Nurses; IC physicians, Infection Control Doctors.

## Data Availability

The data are available from the corresponding author on reasonable request, but are not publicly available because sharing is restricted by the Institutional Review Board approval and informed consent conditions.

## Abbreviations

IC	Infection control
ICNs	Infection control nurses
LTC	Long-term care
LTCHs	Long-term care hospitals
